# Supplementary material for: RIPK1 regulates starvation resistance by modulating aspartate catabolism
Source: Nat Commun. 2021 Oct 22;12:6144. doi: 10.1038/s41467-021-26423-4 (PMC8536712; doi:10.1038/s41467-021-26423-4)
Supplement: Supplementary file 1 — Supplementary Information [file 41467_2021_26423_MOESM1_ESM.pdf]

Supplementary information for

RIPK1 regulates starvation resistance by modulating aspartate catabolism

Xinyu Mei[1,2,4], Yuan Guo[1,3,4], Zhangdan Xie[1,3,4], Yedan Zhong[1], Xiaofen Wu[1,3], Daichao Xu[1], Ying Li[1], Nan Liu[1], Zheng-Jiang Zhu [1,*]

[1] Interdisciplinary Research Center on Biology and Chemistry, Shanghai Institute of Organic Chemistry, Chinese Academy of Sciences, Shanghai, 200032 P. R. China

[2] Center for Clinical Research and Translational Medicine, Yangpu Hospital, Tongji University School of Medicine, Shanghai, P.R. China

[3] University of Chinese Academy of Sciences, Beijing, 100049 P. R. China

[4] These authors contributed equally

[*] Correspondence: jiangzhu@sioc.ac.cn

List of Supplementary Figures

Supplementary Figure 1: The function of RIPK1 in cell survival and mouse postnatal survival (related to Figure 1)

Supplementary Figure 2: Aspartate is a RIPK1-dependent metabolite under starvation (related to Figure 2).

Supplementary Figure 3: Autophagy related genes were not decreased in *Ripk1*$^{-/-}$ cells (related to Figure 3).

Supplementary Figure 4: Increased aspartate induced by RIPK1 deficiency reduced AMP/ATP levels and inhibited AMPK pathway (related to Figure 4).

Supplementary Figure 5: Measurements of ECAR and OCAR with RIPK1 deficiency and aspartate treatment (related to Figure 5).

Supplementary Figure 6: RIPK1 deficiency inhibits aspartate catabolism and autophagy by inactivating SP1 (related to Figure 6).

Supplementary Figure 7: RIPK1 co-located and interacted with SP1 (related to Figure 7).

List of Supplementary Tables

Supplementary Table 1: Gene list for transcription factor prediction in Figure 6d.

Supplementary Table 2: The predicted transcription factors of shared promotor motif of aspartate catabolism genes.

Supplementary Table 3: Detailed information of cell lines in this study.

Supplementary Table 4: Detailed information of recombinant DNA plasmids.

Supplementary Table 5: Detailed information of chemicals and reagents.

Supplementary Table 6: Detailed information of antibodies.

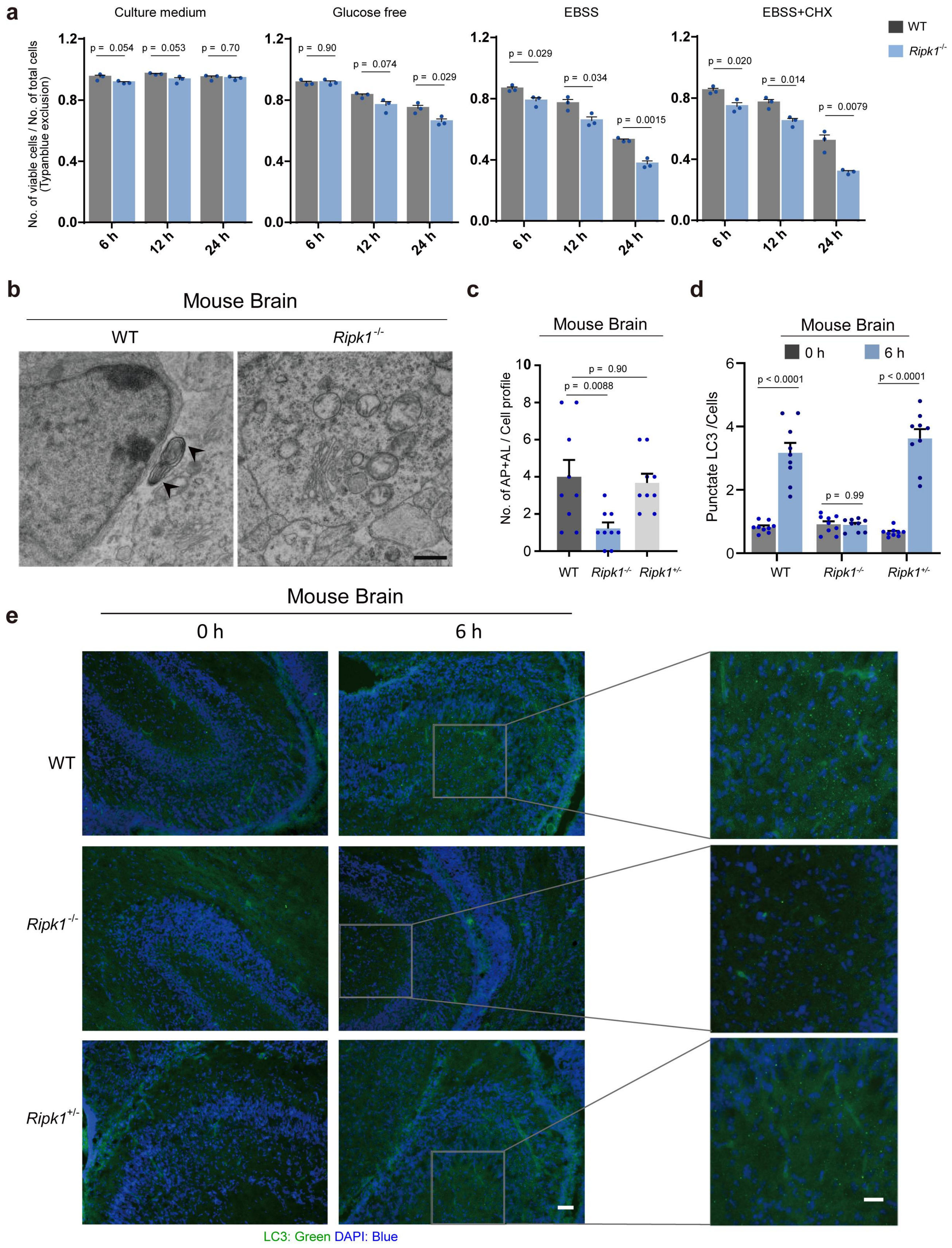
a
Culture medium
Glucose free
EBSS
EBSS+CHX
WT
Ripk1-/-
No. of viable cells / No. of total cells (Typanblue exclusion)
p = 0.054
p = 0.053
p = 0.70
p = 0.90
p = 0.074
p = 0.029
p = 0.029
p = 0.034
p = 0.0015
p = 0.020
p = 0.014
p = 0.0079
6 h
12 h
24 h
b
Mouse Brain
WT
Ripk1-/-
c
Mouse Brain
No. of AP+AL / Cell profile
p = 0.90
p = 0.0088
WT
Ripk1-/-
Ripk1+/-
d
Mouse Brain
0 h
6 h
Punctate LC3 /Cells
p < 0.0001
p = 0.99
p < 0.0001
WT
Ripk1-/-
Ripk1+/-
e
Mouse Brain
0 h
6 h
WT
Ripk1-/-
Ripk1+/-
LC3: Green DAPI: Blue

Supplementary Figure 1. The function of RIPK1 in cell survival and mouse postnatal survival (related to Figure 1)

(a) Assessments of cell survival by trypan blue exclusion. WT and *Ripk1*$^{-/-}$ MEF cells were cultured in culture medium, glucose free medium, EBSS, or EBSS with CHX, respectively, for different times as indicated. Data were expressed as the ratios of viable cell numbers to total cell numbers (n = 3 biologically independent samples per group). P values were determined by Multiple t tests.

(b) Representative TEM images of the brain tissues obtained from WT and *Ripk1*$^{-/-}$ mouse neonates at 6 h after birth. The arrows indicated the formed autophagic vacuoles. Scale bar represents 500 nm.

(c) The numbers of autophagic vacuoles (AP: autophagosome; AL: autolysosome) in TEM images of mouse brain tissues collected from WT, *Ripk1*$^{-/-}$ and *Ripk1*$^{+/-}$ mouse neonates 6 h after birth. Bar results represent mean ± SEM obtained from 9 random cell profiles in each electron microscopy section per experimental group (3 mice per group, 3 images per mouse). P values were determined by one-way ANOVA by Dunnett's multiple comparisons test.

(d) Averaged fluorescent intensities of LC3 dots/cells in immunofluorescence staining images of brain tissues from mouse neonates. Bar results represent mean ± SEM obtained from 9 random cell profiles (3 mice per group, 3 images per mouse). P values were determined by two-way ANOVA by Sidak's multiple comparisons test.

(e) Representative immunofluorescence staining images of brain tissues for LC3 dot intensities (green dots) and DAPI (blue) from WT, *Ripk1*$^{-/-}$ and *Ripk1*$^{+/-}$ mouse neonates at 0 h or 6 h after birth without milk feeding. Scale bar represents 200 μm. Grey boxes indicate the magnified regions. Scale bar in the magnified images represents 50 μm.

Bar graphs represent mean ± SEM.

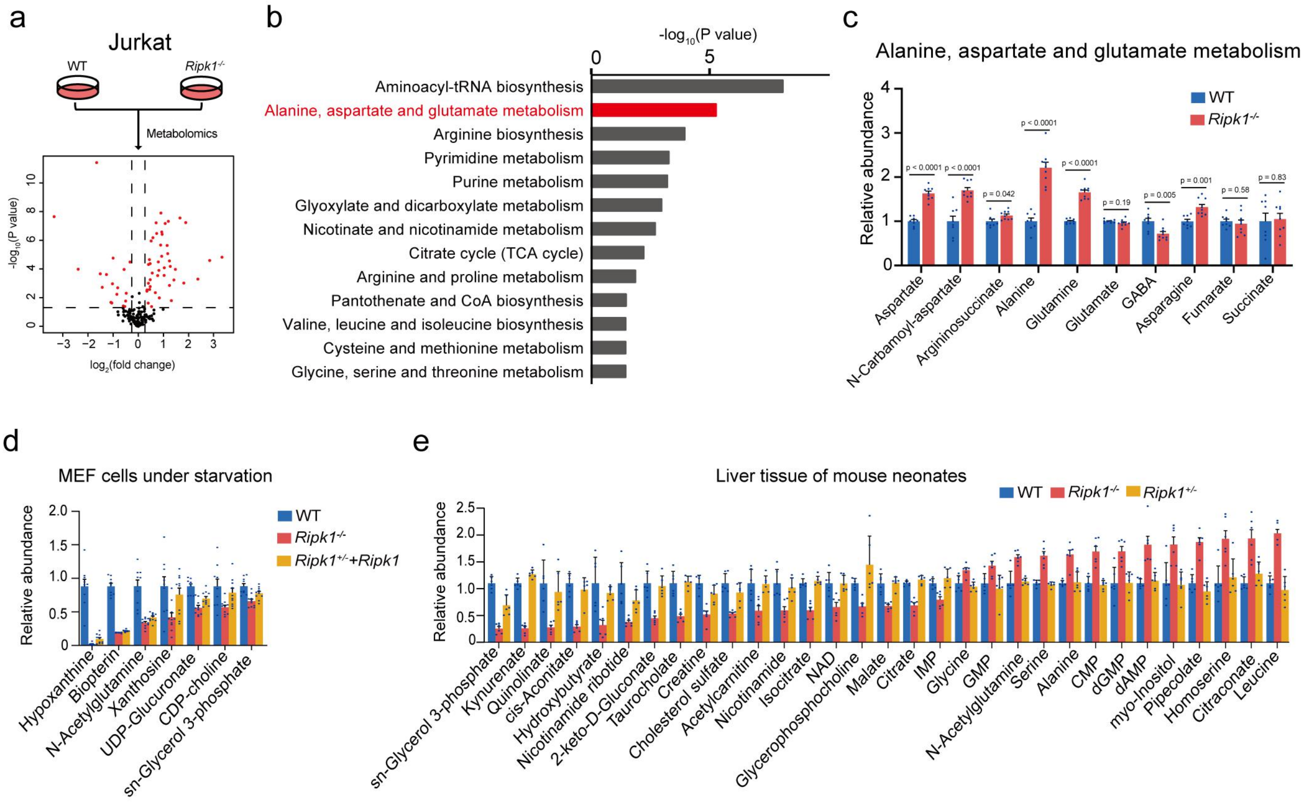

Supplementary Figure 2. Aspartate is a RIPK1-dependent metabolite under starvation (related to Figure 2).

(a) Volcano plot of metabolites in WT and *RIPK1*$^{-/-}$ Jurkat cells. P values were determined by two-tailed Student's t-test. Red dots indicate significantly changed metabolites (p<0.05 and fold change>1.2, WT, n = 8 ; *RIPK1*$^{-/-}$, n = 9 biologically independent samples).

(b) Pathway enrichment analysis of significantly changed metabolites.

(c) Relative abundances of metabolites in the alanine, aspartate and glutamate metabolism pathway were measured in Jurkat cells. WT, n = 8; *Ripk1*$^{-/-}$*,n*= 9 biologically independent samples. P values were determined by two-tailed Student's t-test.

(d) Additional metabolites were significantly increased/decreased in *Ripk1*$^{-/-}$ compared with WT (p<0.05), and significantly rescued (p<0.05) in *Ripk1*$^{-/-}$ + *Ripk1* compared with *Ripk1*$^{-/-}$ in MEFs under starvation condition (EBSS). WT group, n = 9 biologically independent samples; *Ripk1*$^{-/-}$ group, n = 10; *Ripk1*$^{-/-}$ + *Ripk1* group, n = 10 biologically independent samples. P values were determined by two-tailed Student's t-test using adjusted FDR . The data is related to Figure 2d.

(e) Additional metabolites were significantly (p<0.05) increased/decreased in *Ripk1*$^{-/-}$ compared with WT, and significantly rescued (p<0.05) in *Ripk1*$^{+/-}$ compared with *Ripk1*$^{-/-}$ in mouse liver under starvation condition. P values were determined by two-tailed Student's t-test using adjusted FDR (n = 6 biologically independent samples per group). n = 6 biologically independent samples. The data is related to Figure 2f.

Bar graphs represent mean ± SEM.

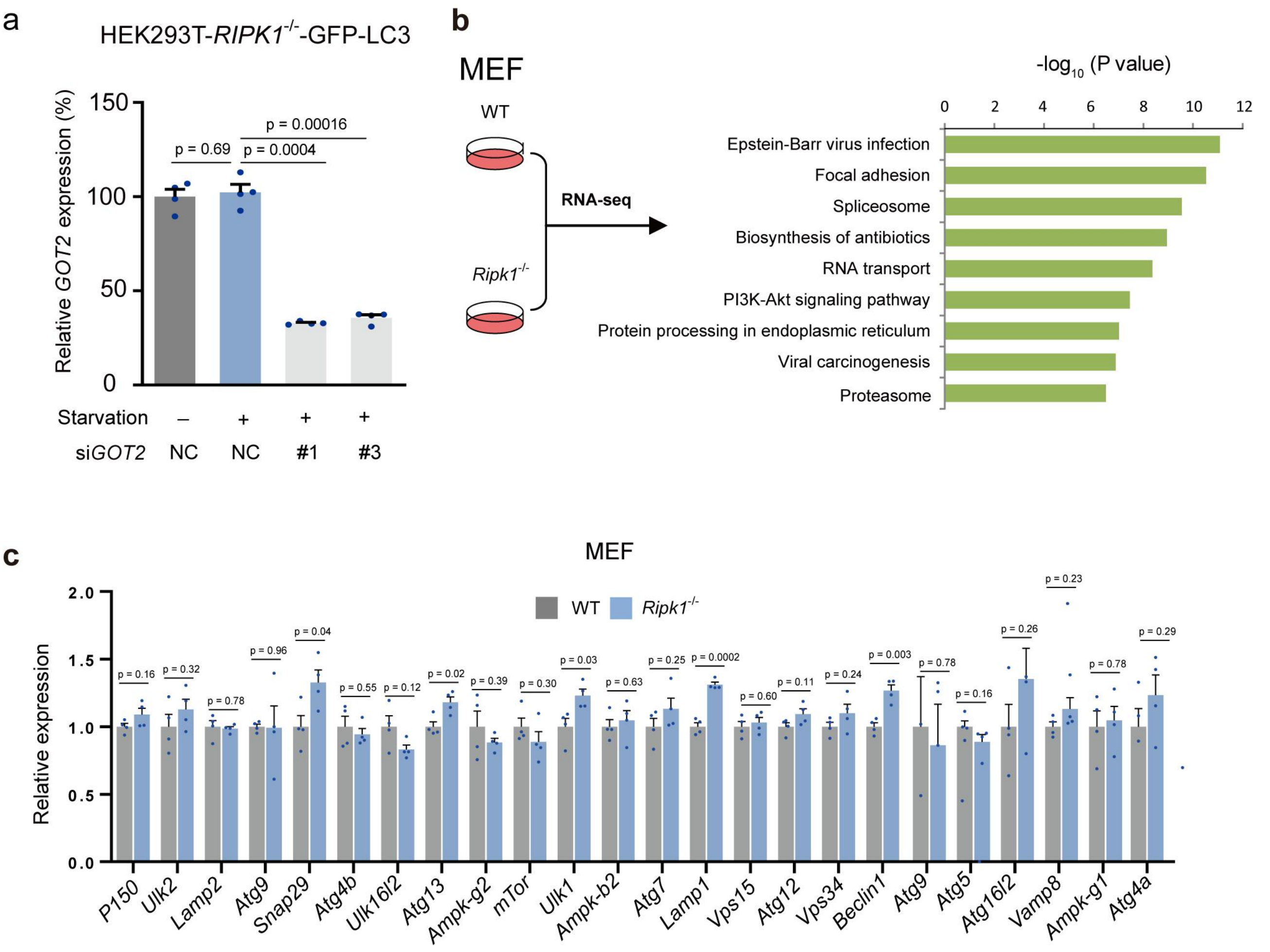

Supplementary Figure 3. Autophagy related genes were not decreased in *Ripk1*$^{-/-}$ cells (related to Figure 3).

(a) Relative expressions of GOT2 mRNA levels were detected by realtime-PCR and normalized relative to the starvation-/NC group. HEK293T cells were transfected with pcDNA3.1-GFP-LC3 plasmids. At 16 h after the transfection, cell passages were divided to 4 groups (3 replications per group) and transfected with NC/siRNAs targeting GOT2. Cells were cultured in culture medium or EBSS as indicated (n = 4 biologically independent samples per group). The data is related to Figure 3h. P values were determined by two-tailed Student's t-test.

(b) Pathway enrichment analysis of significantly changed genes ($p < 0.05$) in WT and *Ripk1*$^{-/-}$ MEFs measured by RNA-seq.

(c) Relative mRNA expression of autophagy related genes in WT and *Ripk1*$^{-/-}$ MEFs measured by RNA-seq (n = 4 biologically independent samples per group). P values were determined by two-tailed Student's t-test.

Bar graphs represent mean ± SEM.

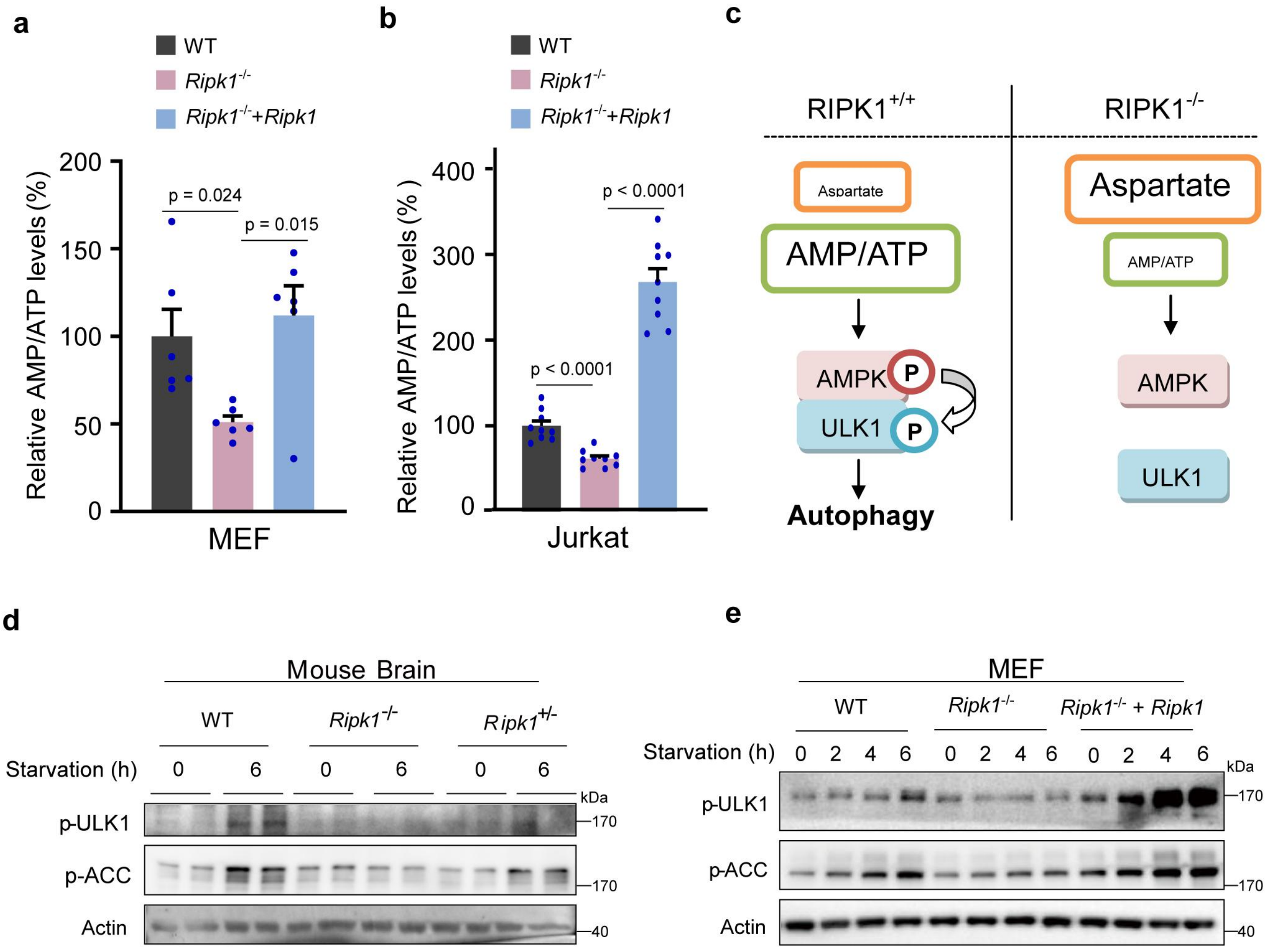

Supplementary Figure 4. Increased aspartate induced by RIPK1 deficiency reduced AMP/ATP levels and inhibited AMPK pathway (related to Figure 4).

(a) Intracellular AMP/ATP levels in WT, *Ripk1*$^{-/-}$ and *Ripk1*$^{-/-}$ + *Ripk1* MEFs were determined by LC-MS (n = 6 biologically independent samples per group). P values were determined by two-tailed Student's t-test.

(b) Intracellular AMP/ATP levels in WT, *RIPK1*$^{-/-}$ and *RIPK1*$^{-/-}$ + *RIPK1* Jurkat cells were determined by LC-MS (n = 9 biologically independent samples per group). P values were determined by two-tailed Student's t-test.

(c) Proposed model for the inhibition of AMPK/ULK1 activation in RIPK1 deficient condition.

(d) Western blot analyses of p-ULK1 (S317) and p-ACC (S79) levels in brain tissues obtained from WT, *Ripk1*$^{-/-}$and *Ripk1*$^{+/-}$ mice without milk feeding. Tissues were harvested at 0 or 6 h after birth as indicated.

(e) Western blot analyses of p-ULK1 (S317) and p-ACC (S79) levels in WT, *Ripk1*$^{-/-}$ and *Ripk1*$^{-/-}$ + *Ripk1* MEFs. Cells were cultured under EBSS condition for 0, 2, 4, or 6 h.

Bar graphs represent mean ± SEM.

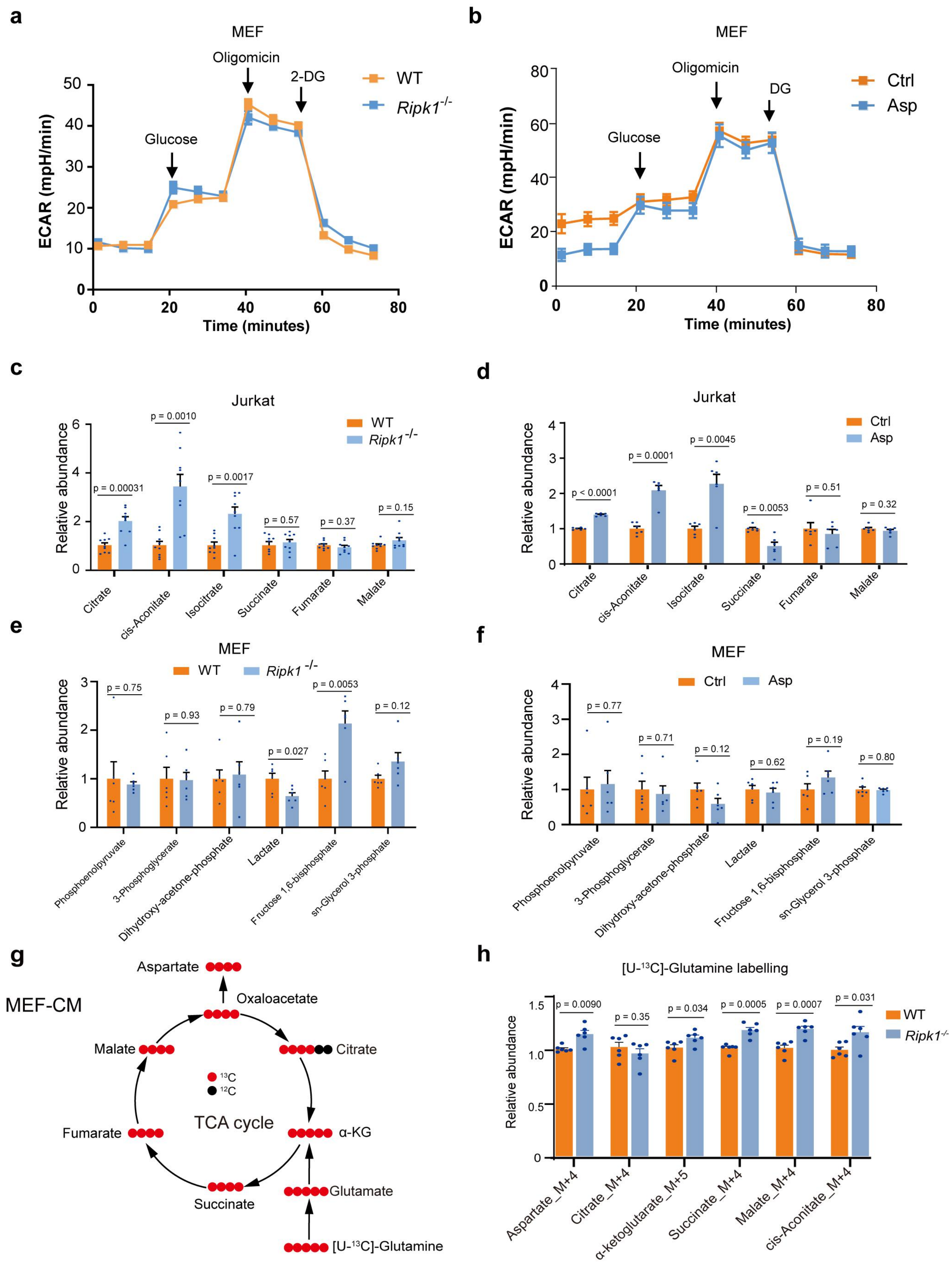
a
MEF
Oligomicin
2-DG
Glucose
WT
Ripk1-/-
ECAR (mpH/min)
Time (minutes)
b
MEF
Oligomicin
DG
Glucose
Ctrl
Asp
ECAR (mpH/min)
Time (minutes)
c
Jurkat
WT
Ripk1-/-
Relative abundance
p = 0.00031
p = 0.0010
p = 0.0017
p = 0.57
p = 0.37
p = 0.15
Citrate
cis-Aconitate
Isocitrate
Succinate
Fumarate
Malate
d
Jurkat
Ctrl
Asp
Relative abundance
p < 0.0001
p = 0.0001
p = 0.0045
p = 0.0053
p = 0.51
p = 0.32
Citrate
cis-Aconitate
Isocitrate
Succinate
Fumarate
Malate
e
MEF
WT
Ripk1 -/-
Relative abundance
p = 0.75
p = 0.93
p = 0.79
p = 0.027
p = 0.0053
p = 0.12
Phosphoenolpyruvate
3-Phosphoglycerate
Dihydroxy-acetone-phosphate
Lactate
Fructose 1,6-bisphosphate
sn-Glycerol 3-phosphate
f
MEF
Ctrl
Asp
Relative abundance
p = 0.77
p = 0.71
p = 0.12
p = 0.62
p = 0.19
p = 0.80
Phosphoenolpyruvate
3-Phosphoglycerate
Dihydroxy-acetone-phosphate
Lactate
Fructose 1,6-bisphosphate
sn-Glycerol 3-phosphate
g
MEF-CM
Aspartate
Oxaloacetate
Malate
Citrate
13C
12C
TCA cycle
Fumarate
α-KG
Succinate
Glutamate
[U-13C]-Glutamine
h
[U-13C]-Glutamine labelling
Relative abundance
p = 0.0090
p = 0.35
p = 0.034
p = 0.0005
p = 0.0007
p = 0.031
WT
Ripk1-/-
Aspartate_M+4
Citrate_M+4
α-ketoglutarate_M+5
Succinate_M+4
Malate_M+4
cis-Aconitate_M+4

Supplementary Figure 5. Measurements of ECAR and OCAR with RIPK1 deficiency and aspartate treatment (related to Figure 5).

(a) Measurements of ECAR of WT and *Ripk1*$^{-/-}$ MEFs. Cells were cultured in EBSS for 4 h before harvest. WT, n = 58 biologically independent samples; *Ripk1*$^{-/-}$*,* n = 34 biologically independent samples, showing mean ± SEM.

(b) Measurements of ECAR of MEFs treated with 0 or 375 μM of aspartate. Ctrl, n =10 biologically independent samples; Asp, n = 12 biologically independent samples, showing mean ± SEM.

(c, d) Relative abundances of metabolites in TCA cycle measured using LC-MS. WT and *Ripk1*$^{-/-}$ Jurkat cells were cultured in EBSS for 4 h before harvest (**c**, n = 9 biologically independent samples per group). WT Jurkat cells were cultured in EBSS supplemented with 0 or 375 μM of aspartate for 4 h before harvest (**d**, n = 6 biologically independent samples per group, showing mean ± SEM). P values were determined by two-tailed Student's t-test.

(e, f) Relative abundances of metabolites in glycolysis pathway measured using LC-MS. WT and *Ripk1*$^{-/-}$ MEFs were cultured in EBSS for 4 h before harvest (**e**, n = 6 biologically independent samples per group). WT MEFs were cultured in EBSS supplemented with 0 or 375 μm aspartate for 4 h before harvest (**f**, n = 6 biologically independent samples per group). P values were determined by two-tailed Student's t-test.

(g) Schematic illustration of stable isotope tracing using [U-^{13}C]-glutamine as a tracer. MEFs were grown in Dulbecco's modified eagle medium (DMEM) without glutamine and supplemented 10% dialysed FBS and 4 mM [U-^{13}C]-glutamine for 12 h before collection. Black dots represent ^{12}C and red dots represent ^{13}C.

(h) Relative abundances of aspartate and other metabolites in TCA cycle after [U-^{13}C]-glutamine treatment of WT and *Ripk1*$^{-/-}$ MEFs for 12 h (n = 6 biologically independent samples per group). P values were determined by two-tailed Student's t-test.

Bar graphs represent mean ± SEM.

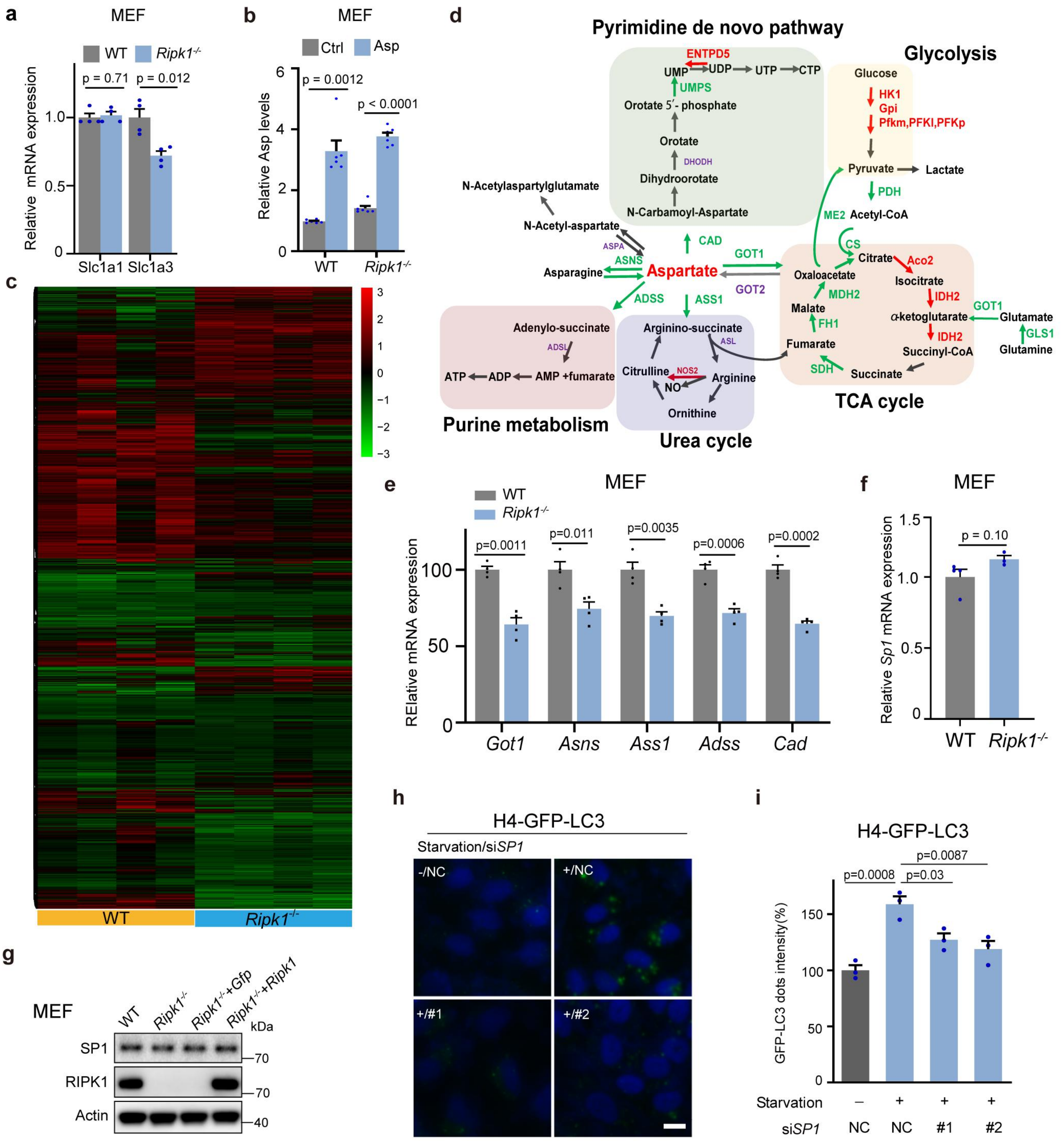
a
MEF
WT
Ripk1-/-
p = 0.71
p = 0.012
Relative mRNA expression
Slc1a1
Slc1a3
b
MEF
Ctrl
Asp
p = 0.0012
p < 0.0001
Relative Asp levels
WT
Ripk1-/-
c
WT
Ripk1-/-
d
Pyrimidine de novo pathway
Glycolysis
ENTPD5
UMP
UDP
UTP
CTP
UMPS
Orotate 5′- phosphate
Orotate
DHODH
Dihydroorotate
N-Carbamoyl-Aspartate
Glucose
HK1
Gpi
Pfkm,PFKl,PFKp
Pyruvate
Lactate
PDH
ME2
Acetyl-CoA
N-Acetylaspartylglutamate
N-Acetyl-aspartate
ASPA
ASNS
Asparagine
Aspartate
CAD
GOT1
GOT2
ADSS
ASS1
CS
Citrate
Aco2
Oxaloacetate
Isocitrate
MDH2
IDH2
Malate
α-ketoglutarate
GOT1
Glutamate
GLS1
Glutamine
FH1
Fumarate
IDH2
Succinyl-CoA
SDH
Succinate
Adenylo-succinate
ADSL
ATP
ADP
AMP +fumarate
Arginino-succinate
ASL
Citrulline
NOS2
NO
Arginine
Ornithine
Purine metabolism
Urea cycle
TCA cycle
e
MEF
WT
Ripk1-/-
p=0.0011
p=0.011
p=0.0035
p=0.0006
p=0.0002
RElative mRNA expression
Got1
Asns
Ass1
Adss
Cad
f
MEF
p = 0.10
Relative Sp1 mRNA expression
WT
Ripk1-/-
g
MEF
WT
Ripk1-/-
Ripk1-/-+Gfp
Ripk1-/-+Ripk1
kDa
SP1
RIPK1
Actin
70
70
40
h
H4-GFP-LC3
Starvation/siSP1
-/NC
+/NC
+/#1
+/#2
i
H4-GFP-LC3
p=0.0087
p=0.0008
p=0.03
GFP-LC3 dots intensity(%)
Starvation
siSP1
NC
NC
#1
#2

Supplementary Figure 6. RIPK1 deficiency inhibits aspartate catabolism and autophagy by inactivating SP1 (related to Figure 6).

(a) Relative mRNA abundances of *Slc1a1* and *Slc1a3* in *Ripk1*$^{-/-}$ MEFs compared to the WT MEFs. n = 4 biologically independent samples per group. P values were determined by two-tailed Student's t-test.

(b) Intracellular aspartate levels in WT and *Ripk1*$^{-/-}$ MEFs responding to 375 μM aspartate treatment were measured by LC-MS (n = 6 biologically independent samples per group). P values were determined by two-tailed Student's t-test.

(c) Heatmap depicting the changes of gene expression in WT and *Ripk1*$^{-/-}$ MEFs measured with RNA-seq.

(d) Changes in the mRNA levels for aspartate catabolic enzymes between WT and *Ripk1*$^{-/-}$ MEFs measured by RNA-seq. Red, up-regulated; green, down-regulated; purple, not significant in *Ripk1*$^{-/-}$ cells compared to the WT group.

(e) Relative mRNA levels of aspartate catabolic enzymes (*Got1, Asns, Ass1, Adss, Cad*) in RNA-seq measurements (n = 4 biologically independent samples per group). P values were determined by two-tailed Student's t-test.

(f) Relative mRNA levels of *Sp1* obtained from RNA-seq (n = 4 biologically independent samples per group). P values were determined by two-tailed Student's t-test.

(g) Western blot analyses of SP1, RIPK1 and Actin in WT, *Ripk1*$^{-/-}$, *Ripk1*$^{-/-}$ +*Gfp* and *Ripk1*$^{-/-}$+*Ripk1* MEFs.

(h) Representative images of autophagosome formation in H4-GFP-LC3 cells in response to the transfection of NC or *SP1* siRNAs (#1 and #2). Cells were cultured in culture medium or EBSS for 4 h before harvest. Scale bar represents 10 μm.

(i) The relative LC3 dot intensities from each condition in (i). Data was measured using an ArrayScan HCS 4.0 reader (n = 3 biologically independent repetitions per group, each point represents mean intensity of 3 images in each repetition). P values were determined by one way ANOVA by Tukey's multiple comparisons t.

Bar graphs represent mean ± SEM.

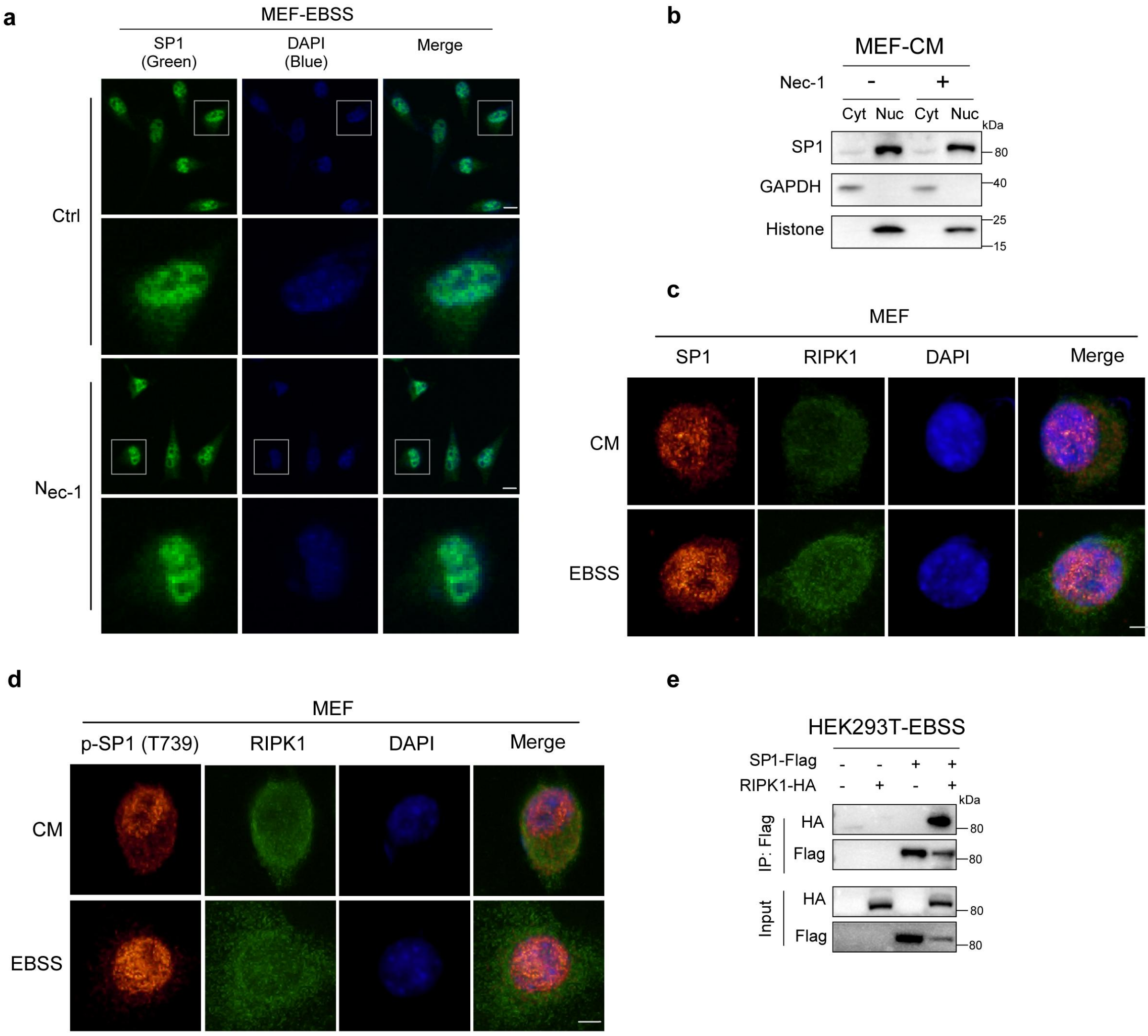

Supplementary Figure 7. RIPK1 co-located and interacted with SP1 (related to Figure 7).

(a) Representative immunofluorescence images depicting the localizations of SP1 (green) in Nec-1 treated or untreated MEFs. Cells were cultured in culture medium (CM) or EBSS for 6 h before collection. The nuclei were stained with DAPI (blue). Scale bar = 10 μm. White boxes indicate the magnified regions.

(b) Cytosol (cyt) and nuclear (nuc) proteins of Nec-1 (100 μM, 24 h) treated or untreated MEF cells were extracted and used to determine SP1 subcellular levels by western blot. Cells were cultured in culture medium (CM).

(c, d) Representative immunofluorescence images depicting the co-localization of RIPK1(green) and SP1 (red, **c**) or p-SP1T739 (red, **d**) in MEFs. Cells were cultured in culture medium (CM) or EBSS for 6 h before collection. The nuclei were stained with DAPI (blue). Scale bar = 5 μm.

(e) Co-immunoprecipitation of SP1 and RIPK1. Flag-*SP1*, HA-*RIPK1* and vector plasmids were transfected in HEK293T cells as indicated. Cells were starved in EBSS for 4 h. Immunoprecipitation was performed using flag-beads.

Supplementary Table 1.

Gene list for transcription factor prediction in Figure 6d.

Gene name	Full name
Got1	Glutamate oxaloacetate transaminase 1
Asns	Asparagine synthetase
Ass1	Argininosuccinate synthetase 1
Adss	Adenylosuccinate-synthetase
Cad	Aspartate transcarbamylase

Supplementary Table 2.

The predicted transcription factors of shared promotor motif of aspartate catabolism genes.

Factor name	MATRIX WIDTH	Start position	End position	String	Dissimilarity
ETF[T00270]	9	0	8	GGGGCGGGG	0
Sp1[T00755]	9	0	8	GGGGCGGGG	0
ADR1[T00011]	8	2	9	GGCGGGGT	0.278687
Spz1[T04668]	7	3	9	GCGGGGT	10.949985
VDR[T00885]	4	6	9	GGGT	0
Zic1[T04669]	4	6	9	GGGT	0
Zic2[T04670]	3	7	9	GGT	0
Zic3[T04671]	4	6	9	GGGT	0
MYBAS1[T05553]	5	0	4	GGGGC	1.224657
MYBAS1[T05553]	5	5	9	GGGGT	0
ZF5[T02349]	3	3	5	GCG	0
E2F-1[T01542]	7	2	8	GGCGGGG	3.397038
E2F-1:DP-1[T05204]	9	1	9	GGGCGGGGT	10.250037
p53[T00671]	7	1	7	GGGCGGG	3.891597
Sp1[T00753]	6	1	6	GGGCGG	0
Sp1[T00752]	10	0	9	GGGGCGGGGT	0.098284
Sp1[T00754]	10	0	9	GGGGCGGGGT	0.590297
Sp3[T02338]	9	0	8	GGGGCGGGG	0
BTEB4[T05053]	9	0	8	GGGGCGGGG	0

Supplementary Table 3.

Detailed information of cell lines in this study

Name	Source	Product No.
MEF	ATCC	BNCC100518
HEK293T	ATCC	CRL-2925
Jurkat	ATCC	BNCC338495
H4-GFP-LC3	Gift from Dr. Daichao Xu lab	NA
Ripk1$^{-/-}$ MEF	Gift from Dr. Junying Yuan lab[1]	NA
Ripk1$^{-/-}$ + *Gfp* MEF	Gift from Dr. Junying Yuan lab[1]	NA
Ripk1$^{-/-}$ + *Ripk1* MEF	Gift from Dr. Junying Yuan lab[1]	NA
Ripk1$^{-/-}$ + *Ripk1*^{K45M} MEF	Gift from Dr. Junying Yuan lab[1]	NA
RIPK1$^{-/-}$ Jurkat	Gift from Dr. Junying Yuan lab[2]	NA
RIPK1$^{-/-}$ + GFP Jurkat	Gift from Dr. Junying Yuan lab[2]	NA
RIPK1$^{-/-}$ + RIPK1 Jurkat	Gift from Dr. Junying Yuan lab[2]	NA
RIPK1$^{-/-}$ HEK293T	Gift from Prof. Jiahuai Han's lab	NA

Supplementary Table 4.

Detailed information of recombinant DNA plasmids

Name	Source	No.
pCDNA3.1-GFP-LC3	Gift from Dr. Daichao Xu lab	NA
pGL3-H_ADSS(-2078 to +315)	Custom synthesized (Genomeditech)	NA
pGL3-H_ASNS(-2106 to +192)	Custom synthesized (Genomeditech)	NA
pGL3-H_ASS1(-1922 to +245)	Custom synthesized (Genomeditech)	NA
pGL3-H_CAD(-2072 to +160)	Custom synthesized (Genomeditech)	NA
pGL3-H_GOT1(-2052 to +196)	Custom synthesized (Genomeditech)	NA
PGL3-basic	Genomeditech	NA
PGMLV-CMV-H_SP1-PGK-Puro Lentivirus	Genomeditech	GM-14025LV
pGM SP1-Luc	Genomeditech	GM-021006
pCMV-RIPK1-HA	Gift from Dr. Daichao Xu lab	NA

Supplementary Table 5.

Detailed information of chemicals and reagents.

Name	Source	Product No.
Aspartate	Sigma-Aldrich	A9978
[U-^{13}C]-glutatmine	Sigma-Aldrich	605166
5-aminoimidazole-4-carboxamide-1-β-D-ribofuranoside (AICAR)	Selleckchem	NSC105823
Aminooxy acetic acid (AOA)	Selleckchem	S4989
2-amino-3-butenoic acid (2-AB)	Dernopharm	DN-XYD-160302-1
Dulbecco's Modified Eagle Medium (DMEM)	Thermo Fisher, GIBCO	11965
Fetal Bovine Serum (FBS)	Thermo Fisher, GIBCO	10099
Penicillin/streptomycin	Thermo Fisher, GIBCO	15140122
Roswell Park Memorial Institute (RPMI) -1640	Thermo Fisher, GIBCO	21870076
EBSS	Thermo Fisher, GIBCO	14155063
NH_4Cl	Sigma-Aldrich	A9434
DAPI	Beyotime	C1005
DAPI	Sigma-Aldrich	D9542
PageRuler Prestained Protein Ladder	Thermo Fisher	SM0671
GoldBand 3-color Regular Range Protein Marker	Yesen	926-98000
Trizol Reagent	Thermo Fisher	15596018
DEPC-treated Water	Thermo Fisher	R0601
PowerUp™ SYBR™ Green Master Mix	Thermo Fisher	A25780
SuperScript™ III First-Strand Synthesis System	Thermo Fisher	18080051
Seahorse XF Glycolysis Stress Test	Agilent	#103020-100
XF Cell Mito Stress Test Kit	Agilent	#103015-100
CellTiter-Glo® Luminescent Cell Viability Assay	Promega	G7570
AMP-Glo Assay Cat	Promega	#V5011
anti-FLAG® M2 Affinity Agarose Gel	Sigma	A2220
Nuclear Protein Extraction kit	Solarbio	R0050

Supplementary Table 6.

Detailed information of antibodies.

Name	Source	Product No.	Dilution
Rabbit anti LC3B	Cell Signaling Technology	#2775	1:1000
Rabbit anti AMPK	Cell Signaling Technology	#2532	1:1000
Rabbit anti phospho-AMPK (Thr172)	Cell Signaling Technology	#2535	1:1000
Rabbit anti ACC	Cell Signaling Technology	#3662	1:1000
Rabbit anti phospho-ACC (Ser79)	Cell Signaling Technology	#3661	1:1000
Rabbit anti SQSTM1/p62	Cell Signaling Technology	#5114	1:1000
Rabbit anti SP1	Cell Signaling Technology	#5931	1:1000
Rabbit anti phospho-SP1 (T739)	Thermo Fisher	PA5-104771	1:500
Rabbit anti RIPK1 (D94C12)	Cell Signaling Technology	#3493	1:1000
Rabbit anti ULK1	Sigma-Aldrich	A7481	1:1000
Rabbit anti phospho-ULK1 (Ser317)	Cell Signaling Technology	#37762	1:1000
Mouse anti Actin	TransGen-Biotech	HC201-02	1:1000
Anti-mouse secondary antibodies	GenScript	A00160	1:5000
Anti-rabbit secondary antibodies	GenScript	A00098	1:3000

References

1. Tao, P. *et al.* A dominant autoinflammatory disease caused by non-cleavable variants of RIPK1. *Nature* **577**, 109-114, doi:10.1038/s41586-019-1830-y (2020).
2. Degterev, A. *et al.* Identification of RIP1 kinase as a specific cellular target of necrostatins. *Nat Chem Biol* **4**, 313-321, doi:10.1038/nchembio.83 (2008).
