# Supplementary material for: RIPK1 regulates starvation resistance by modulating aspartate catabolism
Source: Nat Commun. 2021 Oct 22;12:6144. doi: 10.1038/s41467-021-26423-4 (PMC8536712; doi:10.1038/s41467-021-26423-4)
Supplement: Supplementary file 3 — Description of Additional Supplementary Files [file 41467_2021_26423_MOESM3_ESM.pdf]

Description of Additional Supplementary Files

File name: Supplementary Data 1
Description: The metabolomics data of MEFs under normal condition

File name: Supplementary Data 2
Description: The metabolomics data of Jurkat cells under normal condition

File name: Supplementary Data 3
Description: The metabolomics data of MEFs under starvation condition

File name: Supplementary Data 4
Description: The metabolomics data of Jurkat cells under starvation condition

File name: Supplementary Data 5
Description: The metabolomics data of mouse liver under starvation condition

File name: Supplementary Data 6
Description: The metabolomics data of mouse brain under starvation condition

File name: Supplementary Data 7
Description: The transcriptomics data of MEFs under normal condition

File name: Supplementary Data 8
Description: Detailed information of siRNA and shRNA sequences

File name: Supplementary Data 9
Description: Detailed information of primers sequences
